# Remimazolam Protects Against LPS-Induced Endotoxicity Improving Survival of Endotoxemia Mice

**DOI:** 10.3389/fphar.2021.739603

**Published:** 2021-11-19

**Authors:** Xiaolei Liu, Shaoping Lin, Yiyue Zhong, Jiaojiao Shen, Xuedi Zhang, Shuhua Luo, Li Huang, Liangqing Zhang, Shuangnan Zhou, Jing Tang

**Affiliations:** ^1^ The Department of Anesthesiology, Affiliated Hospital of Guangdong Medical University, Zhanjiang, China; ^2^ Department of Infectious Diseases, Fifth Medical Center of Chinese PLA General Hospital, Beijing, China

**Keywords:** benzodiazepine, endotoxemia, inflammatory, remimazolam, TLR4

## Abstract

Remimazolam is a new benzodiazepine of sedative drugs with an ultra-short-acting anesthetic effect, commonly used for critically ill patients (especially septic patients) in intensive care units (ICUs). Although some anesthetics have been reported to show certain anti-inflammatory effects, the role of remimazolam in inflammation is still remained unknown. Here, we studied the effects of remimazolam on macrophage in response to LPS both *in vivo* and *in vitro*. Interestingly, compared with LPS treatment group, remimazolam remarkably improved survival rate of endotoxemia mice and decreased the release of LPS-induced inflammatory mediators (such as TNF-α, IL-6, and IL-1β). We further found that remimazolam not only inhibited the activation of MAPK signal pathway at 15 min after LPS treatment but also disturbed Rab5a related TLR4 expression at cell surface in response to LPS at a later time. Such evidence suggests that remimazolam might be beneficial to septic patients who are suffering from uncontrolled inflammatory responses.

## Introduction

Sepsis is a severe inflammatory response to infection and remains the primary cause of morbidity and mortality in intensive care unit (ICU) patients worldwide (Drewry et al., 2017). Sepsis usually triggers cell dysfunction and subsequent organ failure, leading to death. Inflammatory factors such as tumor necrosis factor-alpha (TNF-α), interleukin-6 (IL-6), and interleukin-1 (IL-1) act as key regulators of the immune response and play a critical role in the complex pathophysiological mechanisms of sepsis (Parameswaran and Patial, 2010; Schulte et al., 2013). Excessive release of these inflammatory cytokines can lead to dysfunction of the immune system and, consequently, damage to multiple tissues (Schulte et al., 2013). Therefore, coordinating the expression of these inflammatory cytokine genes will be a benefit in the treatment of sepsis. Toll-like receptor (TLR) 4, as one of the most critical receptors for lipopolysaccharide (LPS) induction, has been investigated a lot. Rab5a plays a crucial role in actin remodeling, TLR4-MyD88 interaction, and receptor internalization (Chen et al., 2012; Langemeyer et al., 2018). Early LPS/TLR4 endocytosis mediated by clathrin by Rab5a promotes the transport of more TLR4 stored in the Golgi apparatus to the cell membrane, thus amplifying inflammatory signaling and exacerbating the inflammatory response and cell death (Tang et al., 2020).

Remimazolam is a new ultra-short acting benzodiazepine that is currently under development for intravenous use in procedural sedation and general anesthesia (Wesolowski et al., 2016; Keam, 2020). Remimazolam possesses a fast offset of sedation and faster recovery than seen with currently available drugs of the same class (Kanto, 1985; Kilpatrick et al., 2007; Wang et al., 2020; Zhou et al., 2020). In the ICU, remimazolam is used to manage critically ill patients who require sedation and analgesia for increasing their comfort and collaboration (Chawla et al., 2017), and long-term infusion or higher doses of remimazolam does not accumulate and prolong effects (Wesolowski et al., 2016). Although remimazolam has been gradually used in the clinic, it is not clear whether it has anti-inflammatory effect, and no literature has been reported at present. The anti-inflammatory effects of remimazolam have not been reported, though remimazolam have been applied gradually in clinic practice. It is well known that the binding sites of benzodiazepine exist inside the central nerve system and also in peripheral tissues (Kaynar et al., 2013). Peripheral benzodiazepine receptor (PBR, also called translocator protein 18 kDa, TSPO) is located primarily on the outer membrane of mitochondria in steroid-producing cells (Papadopoulos et al., 2006; Guilarte, 2019). A possible mechanism of remimazolam could be, at least partially, due to blocking the TLR4 signaling pathway after remimazolam binding to the PBR in macrophages.

Here, we studied the effect of remimazolam on the inflammatory response induced by LPS. Our results demonstrated that remimazolam remarkably improved survival rate of LPS-induced endotoxemia mice. We also displayed that the release of LPS-induced inflammatory mediators (such as TNF-α, IL-6, and IL-1β) were restricted by remimazolam *in vivo*. Furthermore, *in vitro*, we found that remimazolam disturb LPS-induced macrophages overactivation process, including inflammatory signal transduction and the cell surface translocation of TLR4, although not via PBR. In addition, remimazolam attenuated the colocalization between early Rab5a and TLR4. Such evidence suggests that remimazolam might be beneficial to septic patients who are suffering uncontrolled inflammatory responses.

## Materials and Methods

### Animals and LPS Induced Endotoxemia

C57BL/6 (WT) mice were purchased from Dien Gene-Tech (Guangzhou, Guangdong, China). All animal programs were reviewed and approved by the Institutional Animal Care and Use Committees of Guangdong Medical University. Eight-week-old mice with a mean body weight of 25 g were used. Endotoxemia model was induced by intraperitoneal injection of LPS (20 mg/kg) (LPS group, n = 28). To evaluate the effect of remimazolam, mice were intraperitoneally injected (16 mg/kg) 30 min before and subcutaneous injected (8 mg/kg) 30 min after LPS with remimazolam (Re + LPS group, n = 28). Mice received only remimazolam were regarded as the Re group (n = 10). All reagents above were dissolved in normal saline at the indicated concentrations. NS was used as the blank vehicle in the mice of the control group. The mice were dislocated 12 h after the LPS injection, and serum and organ samples were collected at the time of execution. Each group had at least three biological replicates *in vivo*.

### Histological Analysis

Liver and lung tissue were fixed overnight with 4% paraformaldehyde at room temperature, embedded in paraffin, and cut into 4 μm sections. The sections were stained with hematoxylin and eosin.

### CCK-8 Assay

Cell Counting Kit-8 (CCK8) assay (Dojindo, Japan) was performed to evaluate cell viability. In brief, RAW264.7 cells or BMDM were inoculated in 96-well plates with a cell density of 0.8 × 10^5^/well for 24 h. Cells were pretreated with indicated concentrations of remimazolam for 20 min and then treated with LPS (1 μg/ml) for 24 h 10μL CCK-8 was added and incubated for another 2 h. Absorbance was measured at OD-450 nm using a spectrophotometer (ThermoFisher, United States).

### Measurement of Cytokines (TNF-α, IL-6, and IL-1β)

TNF-α(88-7324-77), IL-6(88-7064-22), and IL-1β(88-7013-22) Mouse Uncoated ELISA kits (eBioscience, San Diego, CA, United States) were used to calculate TNF-α, IL-6, and IL-1β protein expression by setting a standard curve according to the manufacturer’s instructions. The measurements were standardized with cell numbers *in vitro*.

### Primary Bone Marrow Derived Macrophage Isolation and Culture

The mice were anesthetized with sevoflurane and died of dislocation. After the separation of bilateral femur and tibia, the bone marrow was flushed out with sterile precooling PBS, and the specific culture method was described above (Li et al., 2016a). In brief, after centrifugation, marrow rinse solution was resuspended in complete medium and cultured with 10 ng/ml M-CSF(#315-02, PEPRO TECH, Rocky Hill, United States) for 3 days, then M-CSF was added again to reach 20 ng/ ml, and the culture was mature on 5–7 days.

### Flow Cytometry Analysis

Macrophages collected from peritoneal lavage were labeled with BV421 Rat Anti-Mouse F4/80 (BD PharmingenTM, San Jose, CA, United States) at 4°C for 15 min. For measuring cell surface expression of TLR4, macrophages were stained with PE conjugated anti-mouse CD284 (TLR4) antibody (eBioscience affymetrix, San Diego, CA, United States) for 30 min. For detecting cell death, cells were incubated with PI and Annexin V (BD PharmingenTM, San Jose, CA, United States) for 15 min at room temp, and the double-stained cells were counted as dead cells. A total of 20,000 events were collected and the cells were analyzed using FACScalibur cytometer (BD Biosciences, San Jose, CA, United States). Peritoneal macrophages were identified as F4/80^high^. The final data were analyzed by FlowJo-V10 software (Tree Star, Ashland, OR, United States).

### RNA Extraction and Quantitative Real-Time PCR

Total RNA was isolated from BMDM or RAW264.7 cells using TRIzol RNA Isolation Reagents (TaKaRa, Janpan). Total RNA was reverse-transcribed into cDNA using a PrimeScript RT reagent Kit with gDNA Eraser (RR047A, TaKaRa, Japan). TB Green Premix Ex Taq II (RR820A, TaKaRa, Japan) was used for real-time fluorescent quantitative PCR (RT-PCR) to detect mRNA expression. ABI 7500 system (Applied Biosystems, United States) was used for performing real-time PCR. Primers sequences are in Supplementary information, Supplementary Table S1. The final results were analyzed and calculated according to an equation: 2^–ΔΔCt^ which provided the number of targets that were normalized to an internal reference. Ct value is the number of cycles required to reach the threshold during PCR process. The test was repeated three times for each biological sample.

### Immunofluorescence

RAW264.7 cells were grown in 35 mm specialized confocal dish (NEST, Wuxi, China). They were first fixed in 2% paraformaldehyde for 15 min, then fixed in 4% paraformaldehyde for 10 min, then permeated with 0.5% TritonX-100, followed by blocked with 5% BSA for 30 min at room temp, respectively. Next, the primary antibody was added and incubated with cells at 4°C overnight. After washing the cells with PBS for three times, appropriate fluorescent secondary antibodies were selected, added, and incubated with cells for 1 h at RT in the dark. After washing three times, the nuclei were stained with DAPI and incubated in the dark for 10 min, and then the plates were sealed with anti-fluorescence quenching agent, and the images were observed under a fluorescence microscope and collected.

### Gene Knockdown

Lipofectamine RNAiMAX Transfection Reagent (#13778150, Invitrogen, United States) was transfected with 100 pmol of mouse PBR siRNA or non-specific siRNA (RiboBio, Guangzhou, China) according to the manufacturer’s instructions. Western blot was used to confirmed the knockdown effect of corresponding gene after 48 h transfection.

### Antibodies and Reagents

**Antibodies:** AKT (4691), P38 (8690), ERK1/2 (4695), p-AKT (4060), p-P38 (4511), p-ERK1/2 (4370), IκBα (4814), p-IκBα (2859), p-IKKα/β (2697), NF-κB (8242), Rab5a (46449), horse anti-mouse IgG (H&L) (7076), and goat anti-rabbit IgG (H&L) (7074) were purchased from Cell Signaling Technology (Danvers, MA, United States). IKKβ (ab124957), PBR (ab109497) was from Abcam (CA, United States). Alexa Fluor 488 goat anti-rabbit (A0423) and Alexa Fluor 647 goat anti-mouse (A0473) secondary antibodies were obtained from Beyotime Biotechnology (Shanghai, China). TLR4 (66350-1-Ig), GAPDH (60004-1-Ig), and β-Tubulin (10068-1-AP) were purchased from Protein-Tech (Wuhan, Hubei, China). Lipopolysaccharides(L4391) was obtained from Sigma (Louis, MO, United States), Remimazolam Tosilate for Injection (Remimazolam) were obtained from Hengrui (Jiangsu, China).

### Statistical Analysis

All results were expressed as mean ± SD. Differences between two groups were analyzed using Student t test. One-way ANOVA was used for comparison of multiple groups. Post hoc analysis was made by using Tukey’s post-hoc test following ANOVA. Survival studies were analyzed by Chi-square test. *p* < 0.05 was considered statistically significant. Graphs and figures were made with Graphpad Prism 6.0 (GraphPad software, CA, United States).

## Results

### Remimazolam Improved LPS-Induced Cell Death *In Vitro*

**FIGURE 1:**
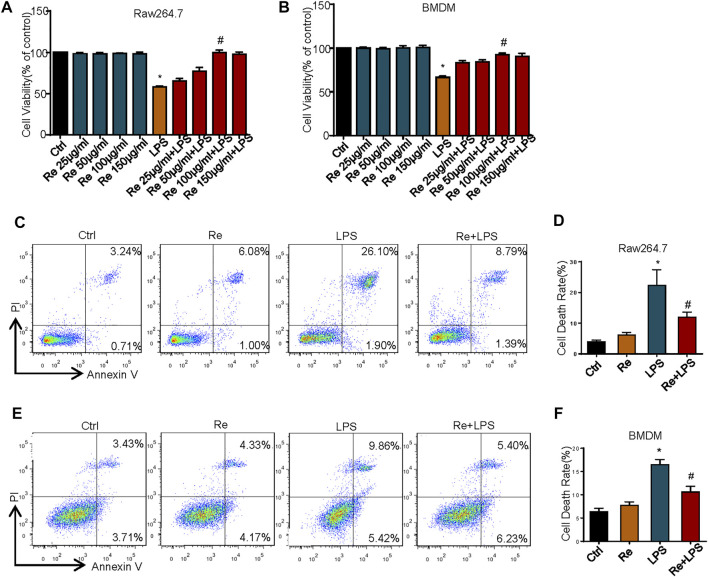
Remimazolam improved LPS-induced cell death *in vitro* and *in vivo*. RAW264.7 cells **(A)** and BMDM **(B)** were exposed to LPS (1 μg/ml, 24 h) with or without different concentration of remimazolam. The CCK8 assay assessed cell viability. **(C,D)** RAW264.7 cells were treated with LPS (1 μg/ml) for 24 h in the presence or absence of pretreatment of remimazolam (Re) for 20 min followed by flow cytometry analysis of cell death. **(E,F)** BMDM were treated with LPS (1 μg/ml) for 24 h in the presence or absence of pretreatment of remimazolam (Re) for 20 min followed by flow cytometry analysis of cell death. Cell death was detected by flow cytometry. All flow cytometric plots were selected as best representatives from at least 3 independent experiments. Each bar represents the mean ± SD, *p < 0.05, compared with negative control group; #*p* < 0.05, compared with LPS alone.

Macrophages are crucial innate immune cells in the body, which play an significant immunoregulatory role in immune homeostasis (Epelman et al., 2014), and also play an essential role in host defense against a variety of pathogens and generation of inflammatory immune response in sepsis (Huen and Cantley, 2017). As a result, we will explore the protective effect of remimazolam on excessive inflammatory injury of the body with macrophages as the research object. To investigate the effect of remimazolam on LPS-induced cell death in macrophages, we first use CCK8 to assess cell vitality. As show in Figures 1A,B, different concentrations of remimazolam alone will not cause the change of cell vitality. At the same time, cell vitality decreases followed by LPS treatment, while pretreatment of remimazolam recovers cell vitality in a concentration-dependent manner. In addition, we also assess cell death by PI and Annexin V by flow cytometry in BMDM and RAW264.7 cells after LPS treatment for 24 h. As shown in Figures 1C–F, LPS increased cell death in RAW264.7 cells and BMDM, and this was attenuated with remimazolam pretreatment. These results suggested that remimazolam was resistant to LPS-induced cell death.

### Remimazolam Inhibits LPS-Induced Inflammatory Response *In Vitro* and *In Vivo*

**FIGURE 2:**
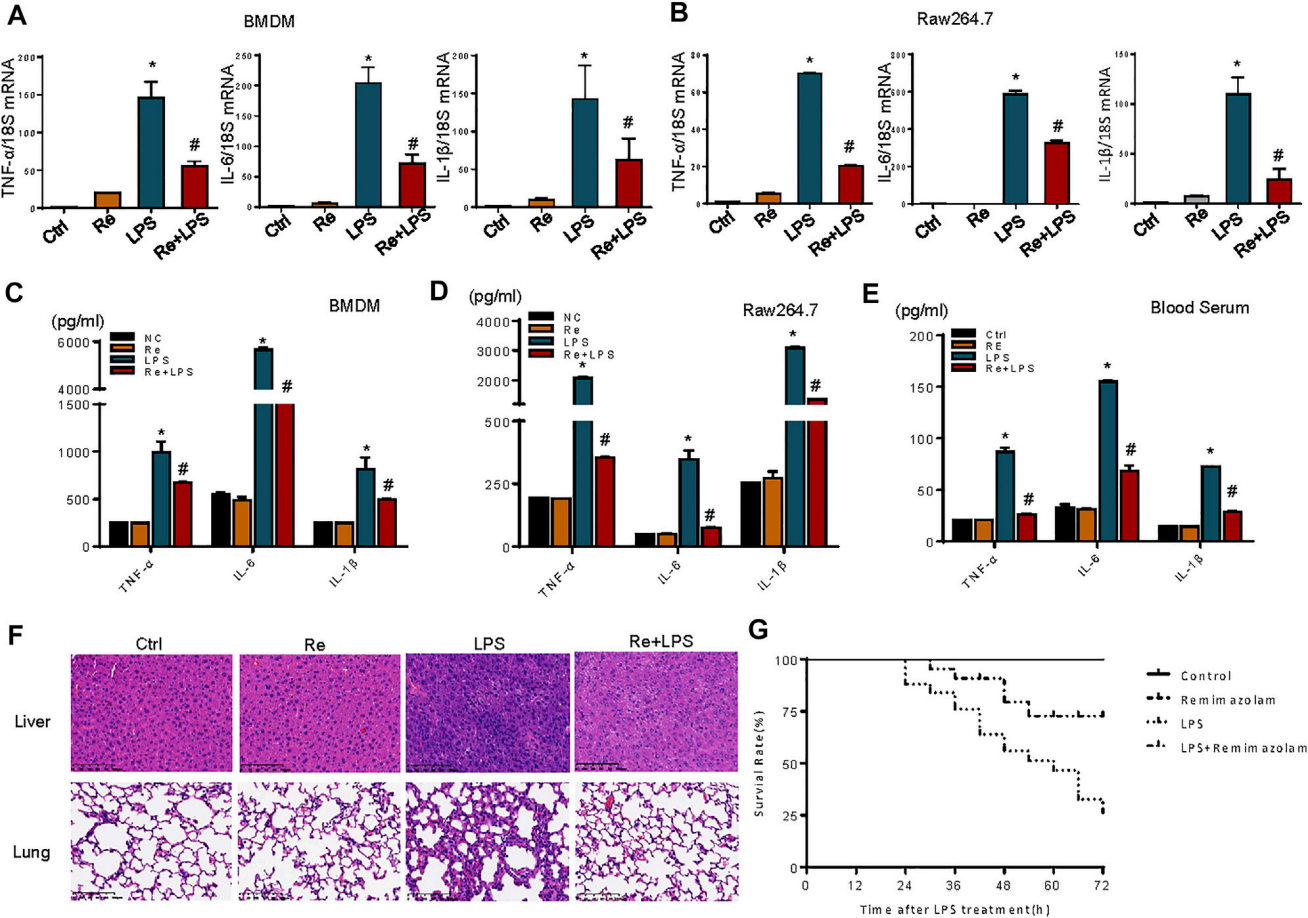
Remimazolam inhibits LPS-induced inflammatory response *in vitro* and *in vivo*. BMDM and RAW 264.7 cells were pretreated with remimazolam 20 min before LPS (1 μg/ml) treatment. **(A)** (BMDM**)**, **(B)** (RAW264.7 cells) Real time PCR analysis of TNF-α, IL-6, and IL-1β mRNA were measured 6 h after LPS treatment. **(C)** (BMDM**)**, **(D)** (RAW264.7 cells) TNF-α, IL-6, and IL-1β proteins were measured in culture medium at 12 h after LPS treatment. Wild type mice were treated in ways as materials and methods. **(E)** TNF-α, IL-6, and IL-1β levels in serum were assayed by ELISA. **(F)** Hematoxylin and eosinstained lung and lung and liver tissue sections. **(G)** After intraperitoneal injection of LPS, survival of mice was detected at every 6 h until 72 h. Survival was significantly increased in LPS + Re group (n = 28) compared to LPS group (n = 28, *p* < 0.05). Each bar represents the mean ± SD. (n = 3), *p < 0.05, compared with negative control group; #*p* < 0.05, compared with LPS alone.

LPS stimulation cause cell inflammatory responses, including increased transcription and expression of inflammatory factors (Foster and Medzhitov, 2009). One of the most dramatic consequences of overwhelming acute inflammation is septic shock due to bacterial LPS, the most potent inducer of the inflammatory response (Hotchkiss and Karl, 2003). For this reason, the effects of remimazolam on LPS-induced macrophage inflammatory response were evaluated. Here, LPS (1 μg/ml) markedly enhanced the mRNA levels of TNF-α, IL-6, and IL-1β, and the phenomenon was partially reversed by remimazolam pretreatment in BMDM and RAW264.7 cells (Figures 2A,B). Subsequently, ELISA was performed to detect inflammatory cytokine expression. As expected, compared with the significantly increased protein levels of inflammatory factors TNF-α, IL-6, and IL-1β in the LPS group, preconditioning with remimazolam reversed the above factor protein expression level, therefore inhibiting the occurrence of inflammation (Figures 2C,D).

We next investigated a potential therapeutic role for remimazolam in LPS-induced endotoxicity *in vivo*. Mice that received an intraperitoneal injection of remimazolam were sedated (lightly anaesthetized), responded to pain, recovered, and began to move about 30 min later. After 12 h of LPS processing (20 mg/kg i. p.), blood serums were collected from mice. Blood serums inflammatory cytokines (TNF-α, IL-6, IL-1β) production from mice after LPS treatment (LPS group) alone were increased, and remimazolam pretreatment (Re + LPS group) markedly prevented LPS-induced production of serums inflammatory factors (Figure 2E). Histological analyses of mouse liver and lung showed that in the control group no inflammation and necrosis were found (Figure 2F). In liver tissue, Re group resulted in spotty coagulation necrosis in the lobules with mild inflammatory cells infiltration within the necrosis area, consisting of neutrophil (the cytoplasm contains fine reddish particles, and the nucleus was divided into leaves) and monocyte (black granular). However, necrosis and inflammation of liver tissue were greatly reduced in Re plus LPS group although the mice received the same dose of LPS. Similarly, in LPS group, the lung tissue displayed serious injury with the features of disrupted alveoli, hemorrhage, thickness of alveolar septum, and infiltration of inflammatory cells. Those pathohistological changes were resolved by treatment with remimazolam. These results revealed that remimazolam prevented the liver and lung injury caused by LPS and protected their function to some extent as well. Meanwhile, the survival rate of control, remimazolam alone, LPS, and LPS plus remimazolam group were studied. As shown in Figure 2G, the survival rate of mice in the LPS group was only 25%, while 75% of mice treated with remimazolam by intraperitoneal injection survived 72 h after LPS administration, much longer than mice injected with LPS alone. Remimazolam alone was not toxic. Collectively, these results indicate that remimazolam significantly prevented multiple organ injury caused by LPS and thus raised survival of endotoxemia mice. In addition, these results give the United States the hint that remimazolam, not just macrophage, may play a potential role to prevent or treat inflammatory diseases in other immune cells.

### Suppressive Effects of Remimazolam on LPS Induced Activation of the MAPK and PI3K Signaling Pathway in RAW264.7 Cells

**FIGURE 3:**
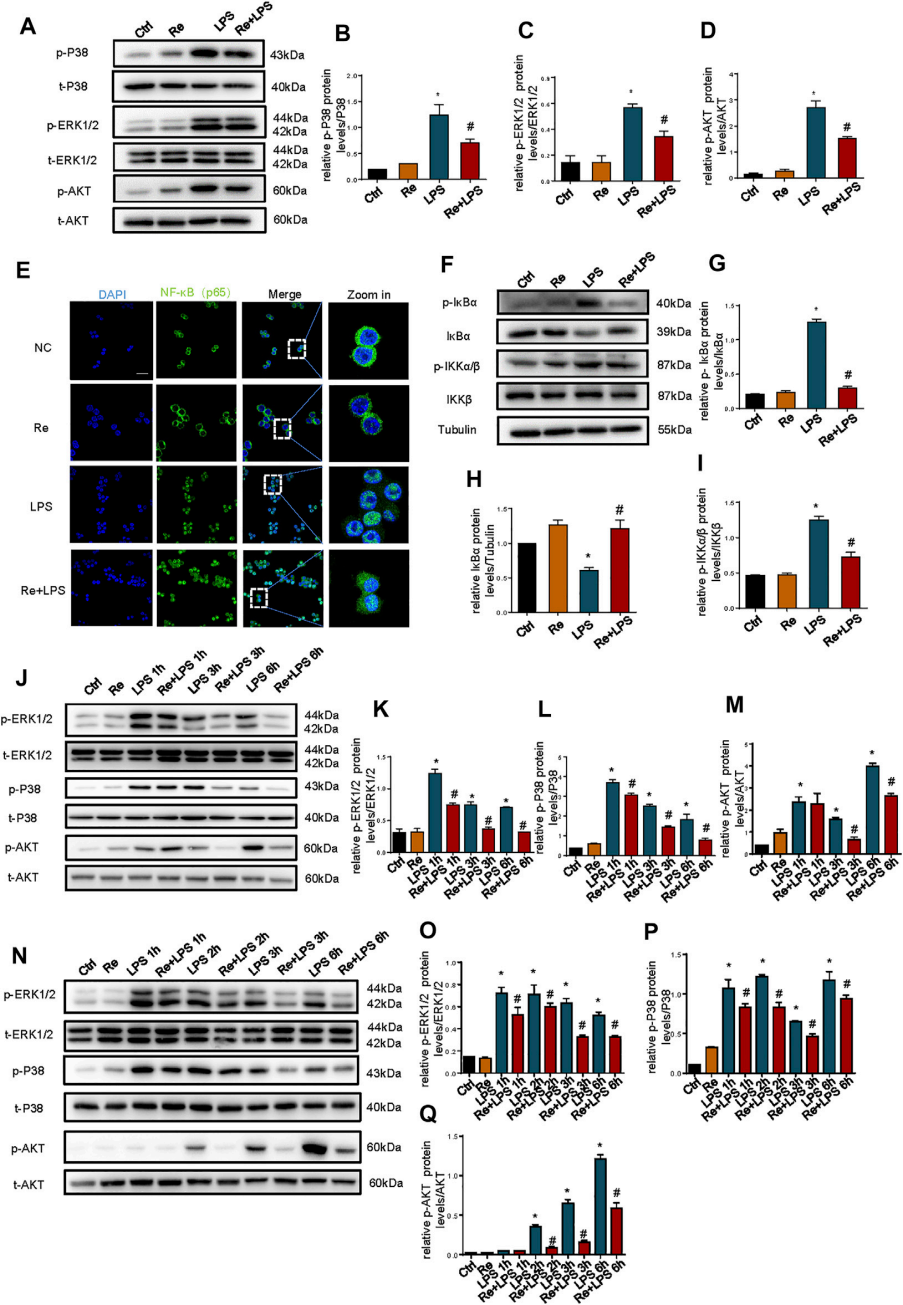
Suppressive effects of remimazolam on LPS induced activation of the MAPK and PI3K signaling pathway in macrophage. Macrophages were treated with LPS (1 μg/ml) with or without pretreatment of remimazolam (Re). **(A)** Western blot analysis of p-P38, P38, p-ERK1/2, ERK1/2, p-AKT, and AKT protein expression were measured 15 min after LPS treatment in RAW264.7 cells. **(E)** Immunofluorescence staining of NF-κB p65 in RAW264.7 were measured 6 h after LPS treatment. **(F)** Western blot analysis of IκBα, p-IκBα, IKKα/β, and p-IKKα/β protein expression were measured 15 min after LPS treatment in RAW264.7 cells. **(G–I)** Protein presence of phosphorylated IκBα, IκBα, and phosphorylated IKKα/β was normalized to IκBα, β-tubulin, IKKα/β respectively. **(J)** Western blot analysis of p-ERK1/2, ERK1/2, p-P38, P38, p-AKT, and AKT protein expression were measured 1, 3, 6 h respectively after LPS treatment in Raw264.7 cells. **N** Western blot analysis of p-ERK1/2, ERK1/2, p-P38, P38, p-AKT and AKT protein expression were measured 1, 2, 3, 6 h respectively after LPS treatment in BMDM. **(B–D)**, **(K–M)**, **(O–Q)** Protein presence of p-ERK1/2, p-P38, and p-AKT was normalized to ERK1/2, P38, and AKT respectively. Each bar represents the mean ± SD. (n = 3), *p < 0.05, compared with negative control group; #*p* < 0.05, compared with LPS group.

MAPK and PI3K signaling pathway, as an upstream signaling pathway of NF-κB, is activated by LPS and is involved in the production of LPS-induced inflammatory cytokines (Barton and Medzhitov, 2003; Ojaniemi et al., 2003). These results show that the phosphorylation of AKT, p38, and ERK1/2 was triggered at 30 min after LPS treatment in RAW264.7 cells, but this activation was substantially suppressed by remimazolam (Figures 3A–D). And then we evaluated the role of NF-κB in the protection of inflammatory response by remimazolam. NF-κB activation is directly related to a sequential cascade, including inhibitor kappa B kinase (IKK)-dependent inhibitor kappa Bα (IκBα) phosphorylation, ubiquitination, and proteolytic degradation, accompanying subsequent translocation of cytosolic NF-κB to the nucleus. As show in Figures 3F,G, LPS significantly upregulated the phosphorylations of IKKα/β and IκBα but degraded IκBα expression in RAW264.7 cells. However, pretreatment with remimazolam reversed these effects, indicating that remimazolam was able to inhibit LPS-induced IKKα/β activation and IκBα degradation. These findings were further supported by immunofluorescent staining showing that remimazolam blocked the nuclear translocation of NF-κB p65 when compared with that of LPS stimulation alone at 10 min (Figure 3E). Taken together, these results elucidate that remimazolam inhibited LPS-induced NF-κB activation.

Furthermore, to determine the effect of remimazolam on advanced inflammatory response, we measured the phosphorylation of AKT, P38, and ERK1/2 after LPS treatment in RAW264.7 cells. ERK1/2, P38, and AKT phosphorylation were measured 1, 3, or 6 h respectively after LPS treatment with or without remimazolam pretreatment. As shown in Figures 3K–M, LPS promoted the phosphorylation of ERK1/2, P38, and AKT at all time points and this effect could be inhibited by remimazolam in 3 or 6 h. Then, we verified this result in BMDM (Figures 3N–Q). Collectively, these results support the notion that the suppressive effect of remimazolam on LPS-induced macrophages activation is through preventing MAPK and PI3K signaling pathway.

### Remimazolam Significantly Suppresses the Macrophage Surface Expression of TLR4 After LPS Treatment

**FIGURE 4:**
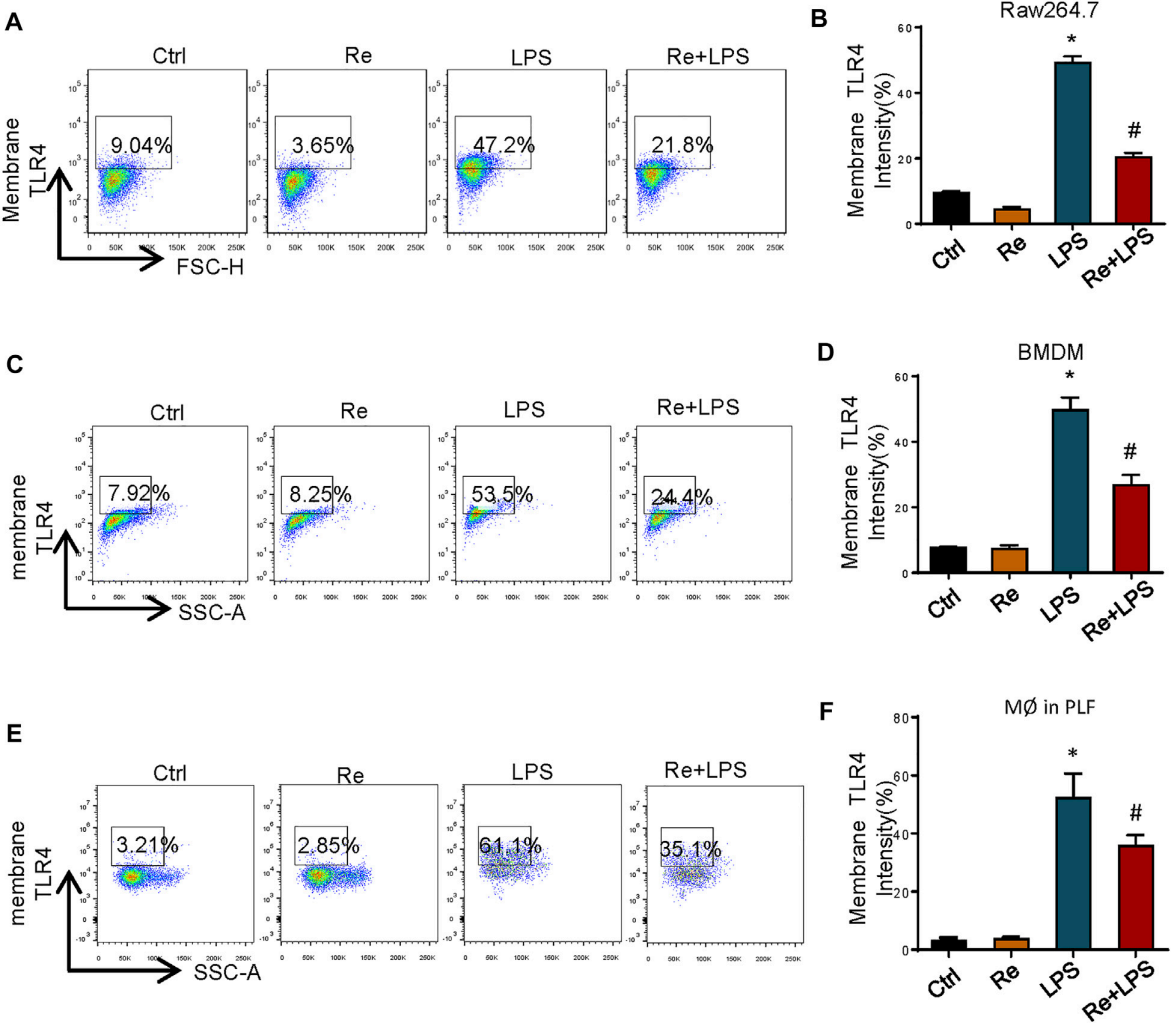
Remimazolam significantly suppresses the macrophage surface expression of TLR4 after LPS treatment. Cells were treated with LPS for 12 h with or without pretreatment of remimazolam (Re) for 20 min. **(A,B)** RAW264.7 cells TLR4 expression was detected by flow cytometry. **(C,D)** BMDM surface TLR4 expression. **(E,F)** Wild type mice were treated in ways as materials and methods. Peritoneal macrophages were collected and stained with F4/80. TLR4 was determined by PE staining. 20,000 events were collected from cells labeled with F4/80. Each bar represents the mean ± SD. (n = 5), *p < 0.05, compared with negative control group; #*p* < 0.05, compared with LPS alone.

TLR4 activation is a tightly regulated process. In addition to directly regulating different signaling pathways, the amount of TLR4/ MD-2 present on the cell surface also controls the LPS response. Based on this, we measured the effect of remimazolam pretreatment in cell surface TLR4 expression after LPS treatment by using flow cytometry. At 12 h after LPS treatment, TLR4 expression on the surface of RAW264.7 cells was increased ∼6 fold as compared with the control group, and this increase could be partially inhibited by remimazolam (Figures 4A,B). This alteration was observed in BMDM as well (Figures 4C,D).

Next, we decided to verify if this change also happened *in vivo*. As shown in Figures 4E,F, where in contract to LPS group, the amount of TLR4 on the surface of peritoneal macrophages from Re plus LPS group was markedly decreased than the former at 12 h after LPS treatment. These findings indicate that remimazolam inhibits LPS-induced upregulation of macrophage surface TLR4 expression.

### Remimazolam Regulate the Cell Surface Expression of TLR4 After LPS Treatment by Affecting Functions and Expressions of Rab5a

**FIGURE 5:**
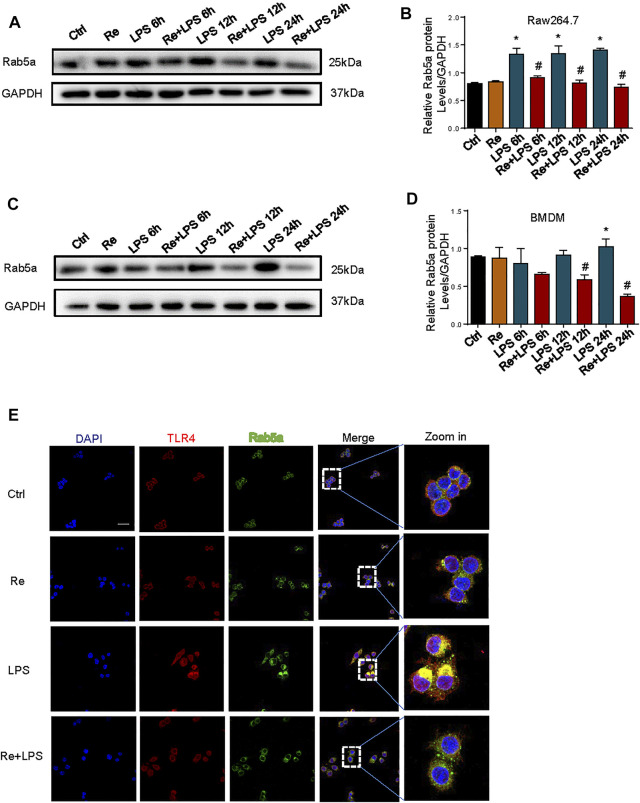
Remimazolam regulate the cell surface expression of TLR4 after LPS treatment by affecting functions and expressions of RAB5a. **(A)** (RAW264.7 cells), **(C)** (BMDM) Western blot analysis of Rab5a expression. **(B–D)** Protein presence of Rab5a was normalized to GAPDH. **(E)** Immune-staining of TLR4 with Rab5a in RAW264.7 cells (up panel; Scale bar, 100 μm). Each bar represents the mean ± SD, *p < 0.05, compared with control group; #*p* < 0.05, compared with LPS group.

Our previous study demonstrated that Rab5a-mediated internalization of TLR4 results in increased cell surface expression of the receptors (Tang et al., 2020). Pretreatment of RAW264.7 cells (Figures 5A,B) and BMDM (Figures 5C,D) with remimazolam given 20 min prior to LPS effectively inhibited the expression of Rab5a at protein level at 6, 12, and 24 h after LPS treatment. We further determined whether remimazolam modulates Rab5a in the progress of blocking macrophage activation. As shown in Figure 5E, remimazolam pretreatment attenuated the colocalization between early Rab5a and TLR4 at 1 h after LPS treatment in RAW264.7 cells by confocal immunoluorescence microscopy. Collectively, these results indicate that remimazolam regulates functions and expressions of Rab5a, and consequently enhanced late phase cell surface expression of TLR4 in response to LPS.

### The Anti-Inflammatory Effect of Remimazolam on Macrophages Was Not Exerted via the PBR

**FIGURE 6:**
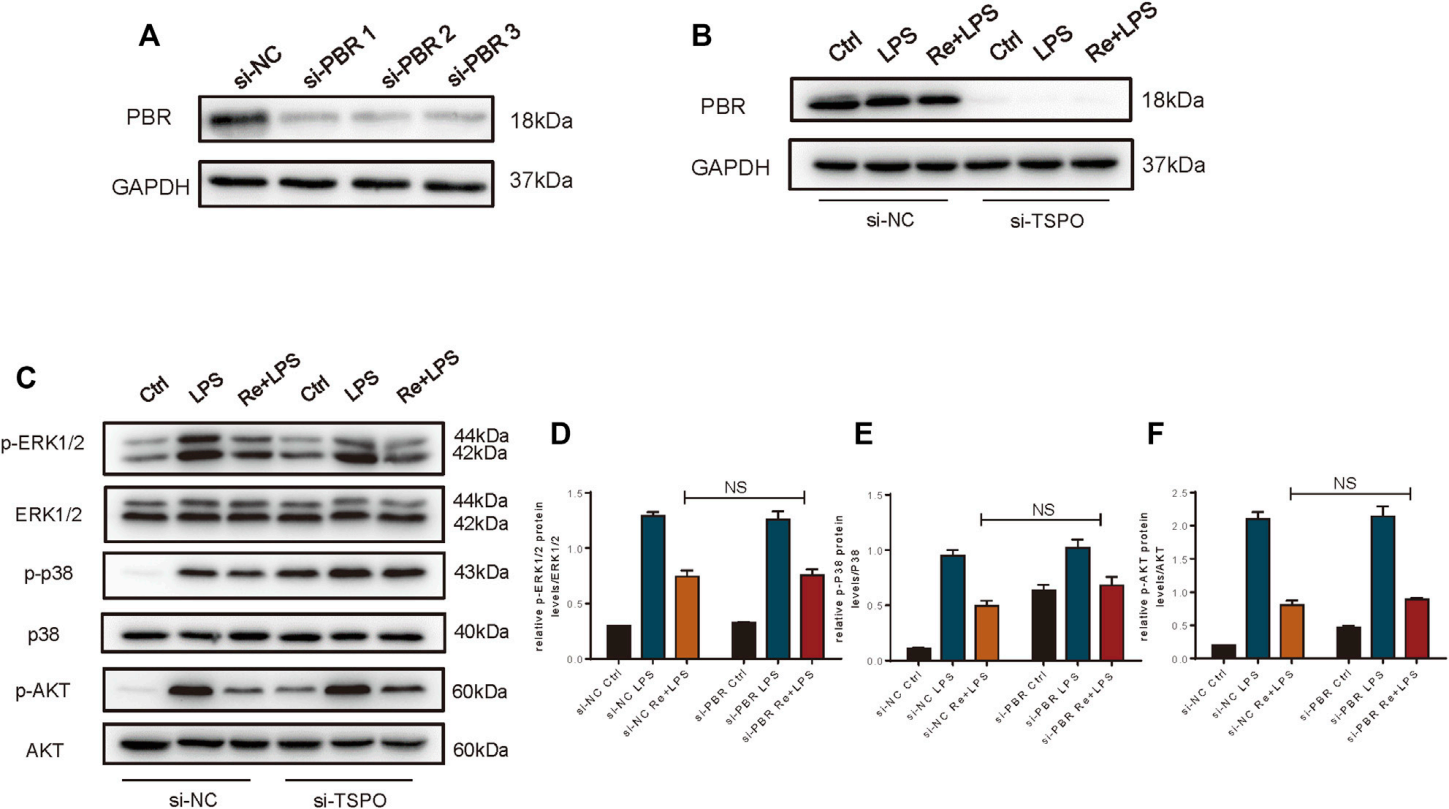
The anti-inflammatory effect of Remimazolam on macrophages was not exerted via the PBR. **(A,B)** Efficiency of si-PBR in Raw265.7 cells. In some groups, RAW264.7 cells were treated with remimazolam (Re) for 20 min or pre-transfected with si-NC, si-TSPO for 48 h before LPS treatment (3 h). **(C)** Western blot analysis of p-ERK1/2, ERK1/2, p-P38, P38, p-AKT, and AKT protein expression. **(D−F)** Protein presence of p-ERK1/2, p-P38, and p-AKT was normalized to ERK1/2, P38, and AKT respectively. Each bar represents the mean ± SD, *p < 0.05, compared with control group; #*p* < 0.05, compared with LPS group; NS no significance.

Therefore, we studied macrophage with PBR knockout. Knockdown efficiency is shown in Figures 6A,B. However, we found that the inhibition of remimazolam on MAPK and PI3K signaling pathway proteins in response to LPS did not reverse after PBR knockout in RAW264.7 cells (Figures 6C–F). These results indicated that remimazolam could not inhibit the inflammatory response by binding PBR in macrophage.

## Discussion

Sepsis represents a dysregulated host response to infection leading to organ dysfunction (Singer et al., 2016). In response to pathogen, immune cells (such as macrophage) are activated to interact with the endothelium through corresponding recognition receptors producing cytokines, proteases, kinins, reactive oxygen species, and nitric oxide (Mahapatra and Heffner, 2021). It is pathogen that triggers an initial exaggerated inflammatory-immune response, leading to activation or suppression of multiple endothelial, hormonal, bioenergetic, metabolic, immune, and other pathways (Arina and Singer, 2021). These, in turn, produce the circulatory and metabolic perturbations resulting in organ dysfunction. Excessive cellular immune response leads to proinflammatory responses and multiple organ damage (Brevetti et al., 2010; Ramachandran, 2014; Cecconi et al., 2018). Therefore, immunotherapy is an important means for the treatment of sepsis, such as anti-inflammatory and immunostimulation therapy (Rubio et al., 2019). Judicious and early antimicrobial administration and early goal-directed therapies have significantly and positively impacted sepsis-related mortality.

Remimazolam is a new sedative drug which combines the properties of two other anesthetic drugs: midazolam and remifentanil. It acts on GABA receptors like midazolam and has organ-independent metabolism like remifentanil (Rogers and McDowell, 2010; Goudra and Singh, 2014). Since it was approved for use by state drug administration on December 2020 in China, Remimazolam is widely applied in general anesthesia and ICU sedation in adults including septic patients. So far there is no study reporting the role of remimazolam on the pathophysiological process of sepsis, except for its sedative effect. Here, we demonstrated that compared with the LPS group, remimazolam effectively decreased LPS induced release of serum pro-inflammatory cytokines and multiple organ damage such as lung and liver. As a consequence, remimazolam treatment dramatically increased the survival rate of endotoxemia mice indicating a potential therapeutic benefit for septic patients. Nevertheless, with regard to the dose of remimazolam, calculated from the ratio of human and animal equivalent doses to body surface area, the dose applied to mice was about nine times that of patients. Since our animal experiments were conducted through intraperitoneal injection, the dose was obtained by observing the state of the mice after injection. We recognized that this was the limitation of the experimental-based study.

LPS is a macromolecular glycolipid in the outer leaflet of the outer membrane of most of the Gram-negative bacteria. It is specifically recognized by transmembrane protein TLR4 which is a member of the toll-like receptor family, leading to inflammatory cytokine production and innate immune system activation (Lakhani and Bogue, 2003). When activated by LPS, TLR4 recruited the adaptor protein myeloid differentiation factor 88 (MyD88), leading to NF-κB and MAPK pathways activation following induction of proinflammatory cytokines (Lakhani and Bogue, 2003; Bode et al., 2012; Lim and Staudt, 2013). In addition, the signal transduction pathway mediated by phosphoinositide 3-kinase (PI3K) and its substrate, protein kinase B (AKT), have attracted extensive attention in cellular inflammatory response (Lakhani and Bogue, 2003; Lim and Staudt, 2013). PI3K/AKT is also involved in LPS-induced cell inflammatory response. Here, we found remimazolam could inhibit the activation of both MAPK and PI3K signal pathway at 15 min after LPS treatment in RAW264.7 cells. This may partially explain why pro-inflammatory cytokines, such as TNF-α, IL-6, and IL-1β, decreased and organ damage was alleviated in the remimazolam pretreated group.

Cell surface expression of TLR4 on innate immune cells critically regulates host responses and progression of inflammation (Parnas et al., 2015). In macrophage, cell surface TLR4 expression is mainly determined by the balance between receptor trafficking from the Golgi apparatus to the cell membrane and internalization of the cell surface receptor into endosomal compartments (Saitoh, 2009). Our previous study revealed that Rab family protein Rab5a is a key molecule mediating the circulation of TLR4 from the cell surface and back in response to LPS. Internalization of TLR4 mediated by Rab5a augment late phase cell surface expression of TLR4 in response to LPS. Therefore, we observed the effect of remimazolam on Rab5a expression and activity. LPS increased the expression of Rab5a both in BMDM and Raw264.7 cells and this effect could be inhibited by remimazolam. We also found that it affects the co-localization of TLR4 and Rab5a in RAW264.7 cells. As a consequence, cell surface expression of TLR4 decreased dramatically in remimazolam plus LPS group. The amount of TLR4 on the cell surface can to some extent determine the intensity of the inflammatory response induced in cells stimulated by LPS. This may be one of the reasons why remimazolam can suppress the activation of P38 and ERK1/2 and the expression of inflammatory cytokines in response to LPS. However, there is no direct evidence that remimazolam can directly regulate TLR4 and thus inhibit these events, and we also speculate whether remimazolam regulates TLR4 through benzodiazepine receptors. In addition to the central nervous system, benzodiazepines action peripheral tissues performed with PBR or mitochondria translocator proteins (Li et al., 2016b). It has been shown that PBR distributes ubiquitously in most types of tissues and was highly expressed in activated macrophages (Vowinckel et al., 1997; Kumar et al., 2010). Therefore, after we knockout the PBR of Raw264.7 cells, we found that the anti-inflammatory effect of remimazolam was not reversed. With a negative outcome, we could conclude that binding to PBR was not responsible for the anti-inflammatory effect of remimazolam on LPS-activated macrophages. Whether remimazolam plays a role by promoting the binding of GABA to the corresponding receptor and increasing the opening time of chloride ion channel after binding with some receptors is worth further exploring. Therefore, we also suspected that remimazolam might be involved in transmembrane transport and ion channels and other immune functions.

**FIGURE 7:**
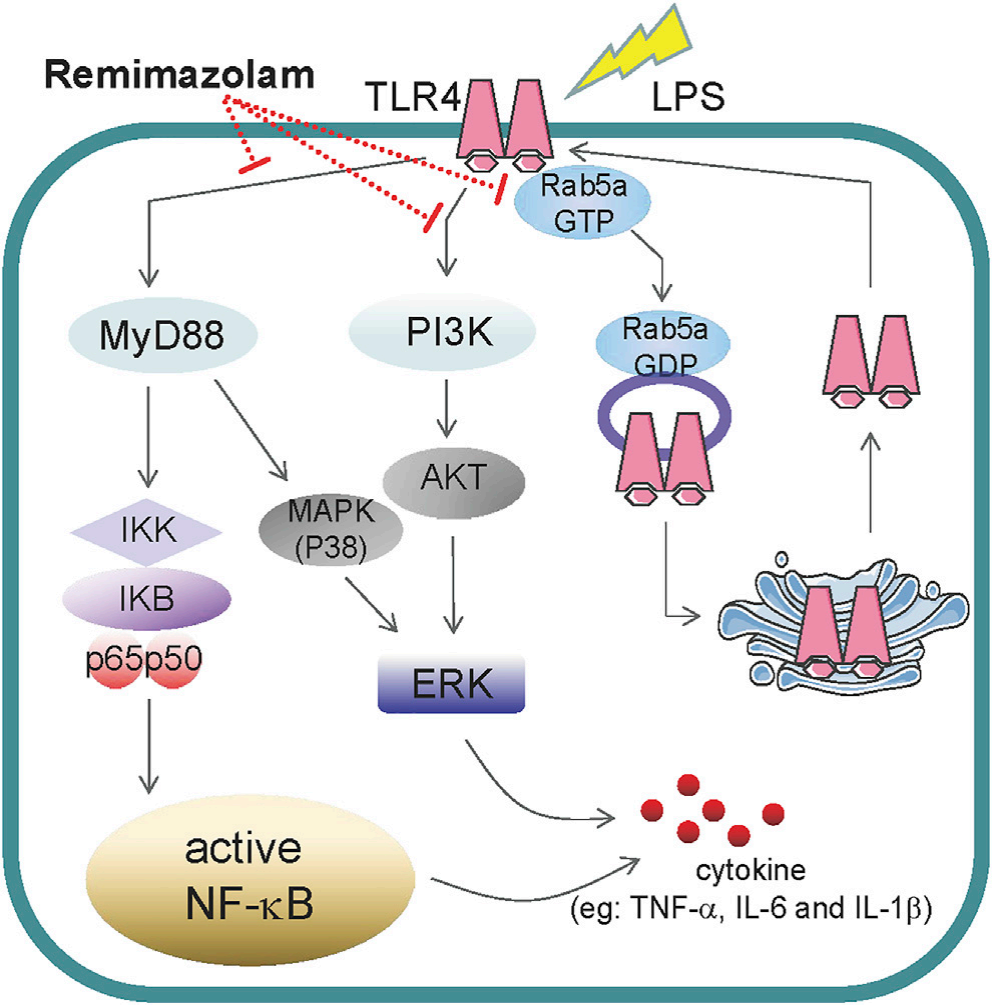
The role of remimazolam in inflammatory. LPS induces macrophage secretion of cytokines, such as TNF-α, IL-6, and IL-1β, which could directly lead to inflammatory response. As a result, remimazolam not only had effect on the activation of NF-κB, p38MAPK, ERK1/2, and PI3K signal pathway at 15 min after LPS treatment but also regulate Rab5a related TLR4 expression at cell surface in response to LPS at later time.

In summary, our study first elucidated that remimazolam can inhibit LPS-induced inflammatory responses of macrophages and obviously improve the survival rate of endotoxemia mice. According to our results, remimazolam suppresses inflammatory response of sepsis at least from two aspects. First, it inhibits the activation of MAPK and PI3K signal pathway at 15 min after LPS treatment. Later remimazolam also disturbs Rab5a related TLR4 expression at cell surface in response to LPS (Figure 7). These results have been verified in RAW264.7 cells and suggest that remimazolam might be beneficial to septic patients who are suffering from uncontrolled inflammatory responses.

## Data Availability

The original contributions presented in the study are included in the article/Supplementary Material, and further inquiries can be directed to the corresponding authors.
