# Supplementary material for: Remimazolam Protects Against LPS-Induced Endotoxicity Improving Survival of Endotoxemia Mice
Source: Front Pharmacol. 2021 Nov 19;12:739603. doi: 10.3389/fphar.2021.739603 (PMC8641375; doi:10.3389/fphar.2021.739603)
Supplement: Supplementary file 1 [file Table1.pdf]

Supplementary Table S1.

	Forward Primer	Reverse Primer
TNF-α	CCCTCACACTCAGATCATCTTCT	GCTACGACGTGGGCTACAG
IL-6	TAGTCCTTCCTACCCCAATTTCC	TTGGTCCTTAGCCACTCCTTC
IL-1β	GCAACTGTTCCTGAACTCAACT	ATCTTTTGGGGTCCGTCAACT
